# Correction

**DOI:** 10.1080/21505594.2026.2697581

**Published:** 2026-07-02

**Article title**: Genetic determinants of hypervirulence with attenuated pathogenicity in a *bla*_NDM-1_-encoding *Klebsiella pneumoniae* causing neonatal sepsis

**Authors**: Deblina Nath, Rinita Dhar, Amrita Bhattacharjee, Sanjib Das, Debadatta Dhar Chanda, Prasanta Kr Borah and Sulagna Basu

**Journal**: *VIRULENCE*

**DOI**: https://doi.org/10.1080/21505594.2026.2679817

When the article was originally published, in Figure 3B, the colours representing the positive control (SB42) and the negative control (ATCC700603) have been interchanged. The corresponding graph lines are reversed. This has now been removed, and the article has been re-published online.

**Figure 3.**
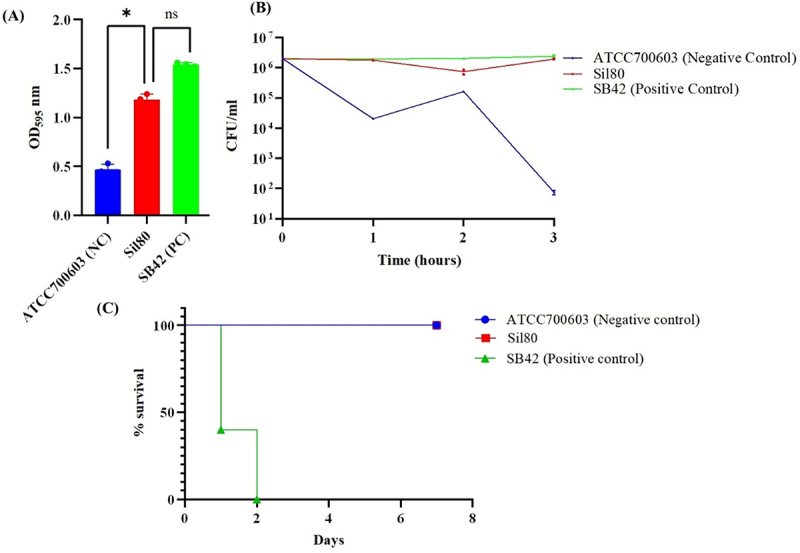
Virulence potential of the study isolate Sil80 (marked in red) compared with ATCC700603 and hvKp-K1 SB42, which were used as negative control (NC) and positive control (PC), respectively, and are marked in blue and green, respectively. (A) The bar graph depicts the quantification of biofilm production by Sil80 using crystal violet staining and absorbance measurement at 595 nm. Sil80 produced significantly higher biofilm levels compared with ATCC700603 (*p < 0.05). (B) The line graph represents the serum bactericidal assay of Sil80. Sil80 showed >100% survival (grade 6 resistance) in human serum. (C) Kaplan–Meier survival curves of Sil80-infected mice. No mortality was observed in mice infected with Sil80, comparable to the ATCC700603 group. Mice in the SB42- infected group showed 100% mortality within two days of infection.

